# HIV preventive behavior scale for Thai men who have sex with men (MSM): development and psychometric properties

**DOI:** 10.12688/f1000research.133299.1

**Published:** 2023-05-18

**Authors:** Passakorn Koomsiri, Nanchatsan Sakunpong

**Affiliations:** 1 Behavioral Science Research Institute, Srinakharinwirot University, Bangkok, 10110, Thailand

**Keywords:** HIV, MSM, Preventive Behavior, Psychometric Properties, Scale

## Abstract

**Background** There are several ways to measure HIV prevention behavior. The simplest is self-assessment. In foreign countries, many scales have been developed. However, there are only a few developed scales among MSM in Thailand and they are not up to date. The objective of this study is to investigate the psychometric features of the HIV preventative behavior measure in Thai men who have sex with men (MSM).

**Methods** The sample consisted of 424 Thai MSM individuals aged 25 or older who had at least one sexual encounter using any method in the previous six months. Test the sample by dividing it in half. Analysis’s construct validity via Exploratory and Confirmatory Factor analysis, reliability using Cronbach’s reliability coefficient. Tests of convergent and discriminant validity based on Pearson correlation coefficients.

**Results** This metric consists of nine items, each comprised of two components: 1) denial and avoidance of the risk of obtaining HIV, and 2) self-protective actions before and during sexual activity. Both components of the CFA were in excellent agreement with the empirical data (χ ^2^ = 36.56, *p* =.06, χ ^2^/df = 1.46, GFI = .96, CFI = .98, AGFI = .94, RMR =.07, RMSEA =.05, TLI = .96). Cronbach’s reliability coefficient is .77, and the HIV Preventive Behavior Scale was significantly linked with the AIDS risk behavior avoidance scale and the AIDS prevention scale ( *r* = .21 and .16, *p*<.01). There was no correlation with the Thai Language Learning Attitude Scale.

**Conclusions** The psychometric qualities are satisfactory and can be used to identify individuals at risk for psychological interventions to enhance HIV preventive behavior among Thai MSM.

## Introduction

Men who engage in sexual relations with other men are regarded as a sexual minority that is persecuted and discriminated against by society. The minority stress model says that men who have sex with other guys are more likely to experience stress due to characteristics that differ from those of the majority. In other words, gender rules that are perceived as alienating have little effect on mainstream society. The resultant bias influences the establishment of minority identities by instilling a sense of alienation, self-loathing, and unacceptability in those who recognize that they have a different gender status and identity than the majority population. This results in a negative self-image, which generates immediate stress and physical or mental health issues. ^1^^,^^2^ The prevalence of sexually transmitted diseases (STDs) was reported to be 35.4% among MSM in Thailand in 2018, with an average sample age of 26 years old. ^3^ In Thailand in 2018, the epidemiological characteristics of HIV infection in men in intimate relationships with men who got treatment and long-term follow-up revealed a prevalence of 15 percent. ^4^ Recent studies reveal that HIV prevalence is high among men who engage in sexual activity with other men. This may be related to the minority stress model described above.

Human Immunodeficiency Virus (HIV) is the virus responsible for acquired immunodeficiency syndromes (AIDS). When it enters the body, it will undergo an incubation period. There may be years without symptoms. After HIV multiplies, it destroys CD4 white blood cells. When white blood cells are depleted, the body lacks immunity, resulting in diseases such as pulmonary tuberculosis and meningitis, as well as fungal infections in various parts of the body, etc. Immunodeficiency syndrome is referred to as AIDS. Patients with advanced AIDS typically die within two to four years if they are not treated early. However, early HIV infection may not necessarily result in AIDS. HIV prevention behaviors should be emphasized in both people living with HIV and those at risk of transmitting the infection to others, including those who are HIV-negative, if the infection has not destroyed the immune system at the onset of various symptoms. ^5^^,^^6^

From the review of concepts related to HIV prevention behaviors, the health belief model concept asserts that perceptions and beliefs of individuals will influence one’s practice to avoid the occurrence of that disease because they believe they are at risk of the disease, have a penalty, and have obstacles that can be severe and affect daily life. Theory of rational action that explains the relationship between attitudes and behavior. ^7^ The notion of self-efficacy and health behaviors that explain an individual’s capacity to control sexual activity related to risky sexual behavior. Low perception of self-control is related to an increased likelihood of engaging in sexual practices that result in HIV infection. ^8^ HIV infection prevention behavior will occur when a person acts or behaves to avoid contracting HIV as because of assessment. A person’s perspective on their odds of contracting HIV and the severity of AIDS, the perceived benefits and hurdles of self-defense, and the presence of a role model or reference group may influence their ability to manage and consistently display HIV prevention practices.

HIV prevention behavior is a health behavior that can be measured or evaluated in a variety of ways, particularly the self-assessment scale at various levels, for which many countries have developed and studied numerous psychological properties, including the AIDS Prevention Questionnaire, the Perceived Risk of HIV Scale, and the Scales to Assess Knowledge, Motivation, and Self-Efficacy for HIV PrEP. ^9^^–^^12^ In Thailand, HIV preventive behavior scale are not being updated. Since 2011, Pimthong and Bhanthumnavin ^13^^,^^14^ have established the AIDS risk behavior avoidance scale and the AIDS preventative behavior scale. Such metrics have been developed for quite some time. ^15^^–^^17^ Therefore, the researcher created a new HIV preventive behavior scale, which led to the purpose of this study, which was to examine the psychometric properties among Thai MSM. This study was conducted within the framework of Thai society by utilizing the cognitive interview technique. Convergent and discriminant validity were also investigated. The researcher believes that this scale can be used to assess the level of HIV prevention behaviors for self-prevention and that multidisciplinary studies can be used as a search engine for sex risk behaviors, leading to counseling or psychological activities to promote and prevent HIV risk behaviors among men who have sex with other men in the future.

## Methods

This study was a cross-sectional study to examination of the psychometric properties of the HIV Prevention Behavior Scale among males who engage in sexual relations with other men. In total, 424 participants responded to an online questionnaire between August and November 2022. On January 19, 2022, the Srinakharinwirot University, Human Research Ethics Committee gave its approval for this study, giving it the approval number SWUEC-G-512/2564E. January 19, 2022.

### Sampling

The subjects are aged 25 years and older, identified themselves as men who had sex with men at least once within the previous six months, and were willing to answer the online HIV preventive behavior scale using a Google form, which required a minimum sample size of 200 based on the Central Limit Theorem, which states that the sample group tends to be normally distributed. ^18^ The Snowball selection sample group was picked, where the sample group introduced a group of persons with comparable qualities and who voluntary selection, obtained a total of 424 samples.

### Instruments

*HIV preventive behavior scale*, consisting of 9 items, was the instrument utilized in this investigation. The response was a five-level self-assessment scale: 1 = never practiced, 2 = rarely practiced, 3 = occasionally practiced, 4 = frequently practiced, and 5 = routinely practiced. ^19^ Average acts or behaviors over the preceding six months. All inquiries were positive. A high total score indicates an elevated level of HIV-preventive behavior. Tool development: From literature studies and research on HIV prevention behavior in Thailand and overseas, instruments were created. The researcher brought the Pimthong and Bhanthumnavin measurements as a model to further build, ^13^^,^^14^ which was developed within the context of the Thai people. First, we are beginning with the development of the model through cognitive interviewing. ^20^ The cognitive interview is a questioning approach based on the respondent’s capacity for recognition and comprehension. This study questioned three specialists with more than five years of expertise in HIV prevention. The substance of the data was evaluated to categorize activities with similar meanings. Afterward, the gathered data was utilized to develop a questionnaire and determine the validity of the content by analyzing the responses. Index of item congruence (IOC) from 3 specialists. ^21^^,^^22^ Including researchers on gender diversity, HIV-related research, and university professors. By eliminating questions with a content validity of less than .60, the items were chosen, with an adjusted item-total correlation below .30. The definition of the entire assessment can be summed up as follows: *"In males who have sex with men and exhibit self-protective actions before and during sex, including effectively responding to or managing sexual emotions."*

*AIDS risk behavior avoidance scale*, developed by Pimthong in 2011, it measures behavioral intent or a person’s readiness to attempt to avoid actions or activities that lead to AIDS risk. By choosing to act or not to act, such as changing sexual partners often taking drugs or intoxicants watching porn, and choosing places that should or should not go, such as entertainment venues, and gay saunas, a total of 12 items, with 7 positive items and 5 negative items. The responses are rated on a 6-point scale ranging from the truest to not true at all. Scores were calculated using a total score of 12-72. If the score was high, it indicated that there was a high risk of AIDS avoidance behavior. For the psychometric characteristics, the discriminant power ranged from 4.74 to 8.93, the item correlation coefficient with the total score was .32 to.64, and the reliability for the whole version with the alpha coefficient was .83. with empirical data with a χ ^2^ = 34.70, df = 46, *p*-value = .89, NFI = .94, GFI = .95, AGFI = .91, SRMR = .049, CFI = 1.00, and RMSEA = 0.0. ^14^

*AIDS prevention scale*, developed by Pimthong in 2011, it is a model to measure a person’s sexual behavior. By considering the method of sexual intercourse, such as using a condom with lubricant every time you have anal sex. Not swallowing partner’s semen, etc. There were a total of 10 items, 4 positive and 6 negative items. Each item had a 6-point evaluation scale ranging from very true to absolutely false by measuring 3 behavioral components. The aspects related to the risk of HIV infection during sex were 1) no risk, 2) moderate risk, and 3) high risk. The score is calculated using a total score of 10-60. If the score is high, it indicates that AIDS prevention behaviors while having sex are high. For the psychometric characteristics, the discriminant power ranged from 4.09 to 9.90, the item correlation coefficient with the total score was .20 to.62, and the reliability for the whole version with the alpha coefficient was .77. with empirical data with a χ ^2^ = 23.52, df = 30, *p*-value = .70, NFI = .93, GFI = .96, AGFI = .92, SRMR = .049, CFI = 1.00, and RMSEA = 0.0. ^14^

*Thai Language Learning Attitude Scale*, developed by Samrongthong in 2011 as a model to measure the attitude towards learning the Thai language. There are a total of 20 items. Answers are self-exploratory. Questions are both positive and negative items. Then choose a response from 5 levels: strongly disagree, disagree, not sure, agree, and strongly agree. There was reliability from internal consistency analysis with Cronbach’s reliability coefficient of .85. ^23^

### Statistical analysis

After acquiring a draft of the HIV Preventive Behavior Scale, its psychometric properties were tested using corrected item-total correlation analysis, and the internal consistency was evaluated using Cronbach’s reliability coefficient. The construct validity was analyzed by exploratory factor analysis (EFA) and confirmatory factor analysis (CFA), including the examination of convergent and discriminant validity by analyzing Pearson product-moment correlation coefficients with the AIDS risk behavior avoidance scale and the AIDS preventative behavior scale, and the Thai Language Learning Attitude Scale.

### Procedures and consent

Data collection for this study was on an online scale. When the sample reads the statement well if they are willing to provide information, their consent was written. The statement will be described the purpose of the data collection, research procedures, and analysis. The collection of data will not identify the identity of the sample.

If the sample does not agree to answer the survey, they can choose to decline to participate in the research and will immediately stop taking the survey. For experts who were cognitive interviewed. The researcher made a letter requesting an interview with the affiliation. The experts can either accept or decline the interview according to their preferences.

## Results

Based on the cognitive interviewing technique, the researcher examined the content and categorized the HIV-preventive activities of men who have sex with males into three broad categories.

*Group 1 Denial and avoidance of HIV risk Behaviors*, is the attempt by men who engage in sexual activity with other men to avoid or reject the stimuli that increase the risk of contracting or transmitting HIV.

*"… avoid holding the gathering outdoors. In the absence of prior acquaintances, when one meets someone, they like, they will poke each other…"* 1st participant.

*"The majority of males who have sex with other men have transient sex partners. There are few consistent sexual partners. Too frequent mate switching poses a risk of illness."* 2nd participant.

*Group 2 Self-protective actions before and during sexual activity*, is the behavior of males who engage in sex with other males to avoid contracting or transmitting HIV before and during sexual activity.

*"If you want to know about protection, you can ask whether or not he used a condom during sexual activity."* 1st participant.

*"Taking drugs during intercourse… will make you inebriated… will cause you to forget to take medications or wear a condom… is an additional factor in the decline of HIV infection."* 3rd participant.

*Group 3 Appropriate sexual response*: in which males who have sex with other guys express their sentiments, sexual emotions, or sexual feelings about themselves or their partners without negatively impacting themselves or others. The promotion of health, spirituality, and self-love

*“… less focus on sexual cues and more focus on other life pleasures… having a mental anchor may reduce sexual preoccupation… ”* 1st participant (further interviews)

*“Various activities, such as aerobic dance, exercising, and running, can be used to reduce sexual desire; this helps… educate people to be safe in love… with acceptable sexual arousal.”* 2nd participant.

Demographic data, the sample used in the study of psychometric properties all 424 people who were men and had sex with a man in the past six months, regardless of their sexual orientation. On average, they were 32.08 ( *SD* = 6.42) years old. IOC was analysed from 3 consensus experts, 19 questions with content validity between .66 and 1.00, then analysed the corrected item-total correlation (CITC) in a sample group of 424 individuals by selecting questions with a value of .30 or greater. ^24^^,^^25^ Skewness and kurtosis were not greater than ±2 when analyzing the normal distribution of the data for the 9 questions with CITC scores between .33-.47, ^18^^,^^26^ which can be utilized to study the data further.

### Construct validity

The researcher used a sample of 424 people to look at the Pearson product-moment correlation coefficients between all nine questions. Each question was found to have a statistically significant association with 35 pairs at the .05 level, at .11-.58, but no higher than .90. ^27^ There was only one set of uncorrelated questions between items 2 and 4. Kaiser-Meyer-Olkin Evaluation of Sampling Efficacy (KMO) = .78, which is close to one and indicates that the model explains 78% of the variance for Bartlett’s Test of Sphericity; χ ^2^ = 843.73, df = 36, sig. = .00, ^28^ indicating that the correlation matrix is not equal to zero. Consequently, it can be studied further for EFA and CFA.

Principal Component Analysis with Varimax and Kaiser Normalization-based EFA results from the first half of a sample of 212 individuals (Convergence occurred after 5 cycles of rotation.) The HIV Preventive Behavior Scale has three Eigenvalues greater than 1 after five rounds ^29^ in three components. The first component was denial and avoidance of HIV risk. The factor weight (factor loading) for each of the three items ranged from .75 to .83. ^30^ Component 2 consisted of self-protective conduct before and during sexual contact. Consisting of items 5 through 9, the weight of each element ranges from .46 to .73. ^30^ However, just item 4 was categorized independently in component 3. Therefore, the number of items is insufficient to be categorized as component 3, ^31^ and after examining the considerate content of item 4, it is determined that it overlaps with component 2, so it is suggested that such questions be included in component 2. As demonstrated in Table 1, the researcher concludes that this measure comprises only two components: denial and risk avoidance of HIV infection and self-protective conduct before and during sexual activity.

**Table 1.** Factor loading, Eigenvalues, % of Variance, and Cumulative % (n=212).

Items	Component 1	Component 2	Component 3
Item 1 You avoid having sexual contact. When it is discovered that your partner is not protected by wearing a condom. คุณหลีกเลี่ยงที่จะมีเพศสัมพันธ์ เมื่อพบว่าคู่นอนของตนไม่ป้องกันด้วยการสวมถุงยางอนามัย	.75		
Item 2 You decline when a buddy invites you to a potentially risky sex activity, such as group sex or traveling to a meeting spot for sex. Substance Dependency. คุณปฏิเสธเมื่อเพื่อนชวนไปร่วมกิจกรรมที่อาจมีความเสี่ยงต่อการมีเพศสัมพันธ์ที่ไม่ปลอดภัย เช่น เซ็กส์หมู่ การไปแหล่งนัดพบเพื่อมีเพศสัมพันธ์ การใช้สารเสพติด	.83		
Item 3 You consistently refuse to switch partners. คุณปฏิเสธการเปลี่ยนคู่บ่อย ๆ	.76		
Item 4 You use a condom during sexual activity. คุณใช้ถุงยางอนามัยเมื่อมีเพศสัมพันธ์			.89
Item 5 You use oil-free lubricants during sexual activity. คุณใช้สารหล่อลื่นที่ไม่มีส่วนผสมของน้ำมันในขณะมีเพศสัมพันธ์		.46	
Item 6 You avoid touching the wound immediately after sexual activity. คุณพยายามไม่สัมผัสบาดแผลโดยตรงหลังจากมีเพศสัมพันธ์		.54	
Item 7 When you need to, you can show your sexual feelings in many ways, such as masturbation, spiritual activities, and recreational activities. เมื่อมีความต้องการ คุณระบายความรู้สึกทางเพศด้วยวิธีการต่าง ๆ ได้ เช่น การสำเร็จความใคร่ด้วยตนเอง กิจกรรมเสริมสร้างจิตวิญญาณ กิจกรรมนันทนาการ		.59	
Item 8 You engage in sexual action while avoiding being harmed or producing blood or lymph. คุณมีเพศสัมพันธ์โดยพยายามไม่ทำให้เกิดบาลแผล เลือด น้ำเหลือง		.66	
Item 9 You engage in sexual activity without causing physical harm to your partner. คุณมีเพศสัมพันธ์โดยไม่ใช้ความรุนแรงด้านร่างกายที่จะทำให้เ กิดบาดแผลกับคู่นอน		.73	
Eigenvalues	2.61	1.49	1.01
% of Variance	29.01	16.60	11.30
Cumulative %	29.01	45.60	56.90

**Figure 1.**
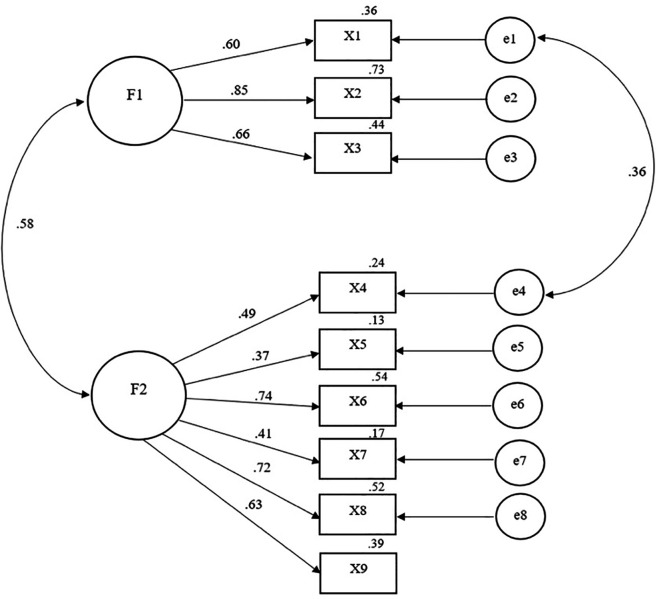
Two-factor CFA of the HIV Preventive Behavior Scale.

The second half of the 212 participants were utilized to conduct a confirmatory factor analysis to validate the HIV Preventive Behavior Scale against empirical data. The two-component confirmation analysis demonstrated that the model was in good agreement with the empirical data. Component 1: denying and avoiding HIV risk consisted of questions 1-3 with factor loading ( *b*); each question is between .60 to .85, ^30^ the standard error ( *SE*) is between .15 to .20, the statistically significant test values ( *t*) are between 7.32 and 7.55 with statistical significance ( *p* < .01), and the coefficient of determination ( *R* ^2^) in each item ranges from .36 to 73, and component 2 is self-protective conduct before and during sexual contact consisted of questions 4-9 ( *b* = .41-74; *SE* = .17-.26; *t* = 4.15-6.29; *p* < .01; *R* ^2^ = .13-.54). Including have factor correlation between component 1 and 2 at .58, see Table 2 (also see Figure 1).

**Table 2.** Factor loading, Standard Error, t Values, Coefficient of Determination by CFA (n = 212).

Items	*b*	*SE*	*t*	*R* ^2^
**Dimension 1, Denying and avoiding the risk of contracting HIV (F1)**				
Item 1 You avoid having sexual contact. When it is discovered that your partner is not protected by wearing a condom. (X1)	.60	-	-	.36
Item 2 You decline when a buddy invites you to a potentially risky sex activity, such as group sex or traveling to a meeting spot for sex. Substance Dependency. (X2)	.85	.20	7.55 **	.73
Item 3 You consistently refuse to switch partners. (X3)	.66	.15	7.32 **	.44
**Dimension 2, Self-protective behavior before and during sex (F2)**				
Item 4 You use a condom during sexual activity. (X4)	.49	-	-	.24
Item 5 You use oil-free lubricants during sexual activity. (X5)	.37	.21	4.15 **	.13
Item 6 You avoid touching the wound immediately after sexual activity. (X6)	.74	.26	6.29 **	.54
Item 7 When you need to, you can show your sexual feelings in many ways, such as masturbation, spiritual activities, and recreational activities. (X7)	.41	.17	4.51 **	.17
Item 8 You engage in sexual action while avoiding being harmed or producing blood or lymph. (X8)	.72	.26	6.26 **	.52
Item 9 You engage in sexual activity without causing physical harm to your partner. (X9)	.63	.26	5.88 **	.39

** *p* < .01.

Consistent with empirical data, the following two components of the HIV Preventive Behavior scale model were identified by CFA: Chi-square: χ ^2^ = 36.56, *p *= .06, Relative Chi-square: χ ^2^/df = 1.46, Goodness of Fit Index: GFI = .9, 6, Comparative Fit Index: CFI = .8, Adjusted goodness of fit index: AGFI = .94, Root Mean Square Residual: RMR = .07, Root Mean Square Error of Approximation: RMSEA = .05, Root mean square residual: TLI = .96. Overall, the model matches the empirical data well (good fit). ^27^^,^^32^^,^^33^

### Reliability, convergent, and discriminant validity

Internal consistency from Cronbach’s reliability coefficient was used to determine that the 9-item HIV Preventive Behavior Scale’s reliability was equivalent to .77 Pearson product-moment correlation coefficients, which were used to examine convergent and discriminant validity among 424 individuals Thai MSM. There are positive correlations between the HIV Preventive Behavior Scale (SUMPHIV) with the AIDS risk behavior avoidance scale (SUML), and the AIDS prevention scale (SUMA) were statistically significant at the .01 level, with correlations of .21 and .26, respectively. There was no correlation between the HIV Preventive Behavior Scale and the Thai Language Learning Attitude Scale (SUMT) (see Table 3).

**Table 3.** Relationship between HIV Preventive Behavior Scale and AIDS Risk Scale, AIDS prevention measure, and attitude measure toward learning the Thai language (n = 424).

Scales	SUMPHIV	SUML	SUMA	SUMT
**SUMPHIV**	-	21 **	.26 **	.08
**SUML**		-	.25 **	.16 **
**SUMA**			-	.18 **
**SUMT**				-
Mean	32.21	43.40	36.44	70.65
Standard deviation	6.39	7.09	6.28	17.43

** *p* < .01.

## Discussion

The purpose of this study was to examine the psychometric properties of the HIV prevention behavior test in Thai MSM. It was determined that this measure had two elements: 1) denial and avoidance of HIV risk and 2) self-protective conduct before and during sexual activity, like previous studies by Pimthong and Bhanthumnavin. ^13^ It was discovered that the AIDS-preventive behaviors of males who engage in sex with other men can be separated into two categories: risk avoidance of HIV infection and self-protective conduct during sex. According to the CFA analysis, nine items of the HIV Preventive Behavior Scale were compatible with empirical evidence. ^9^^,^^10^^,^^34^ χ ^2^ = 36.56, *p* = .06, χ ^2^/df = 1.46, GFI = .96, CFI = .98, AGFI = .94, RMR = .07, RMSEA =.05, TLI = .96. ^27^^,^^32^^,^^33^ The component factor correlation was moderate, with a value of .58 and not exceeding .85, indicating discriminating validity ^35^ and that the components were separated. This study’s findings are comparable to the Two-Dimensional Sexual Sensation Seeking Scale of Ferrer-Urbina *et al.*, 2020, who believed that risky sexual behaviors have been associated with sexual sensation seeking. The researcher and colleagues developed the Sexual Sensation Seeking Scale with nine questions and two components: 1) sexual emotion seeking and 2) sexual boredom. The first section of the questionnaire concerned dangerous behavior, while the second section addressed sexual feelings. There were a total of 770 Latin American adolescents and adults enrolled in the study. Using CFA and ESEM, the researchers determined that structure delivers high levels of reliability and validity, depending on the internal structure of the test. ^36^ It is also consistent with a two-component HIV-related behavioral scale, such as Mena-Chamorro *et al.*’s ^11^ development and proof of the validity of the HIV risk perception scale for young adults in a Hispanic American context. A sample of 524 participants, ages 18 to 33, comprised 49% men and 49% women. 51.84% of those polled identified as heterosexual. There were nine components on the scale. The results indicated that the scale was divided into two components: perceived risk susceptibility and perceived risk severity; however, the focus of this study component is on the perceived risk of HIV infection, and the 2022 study by Sao *et al.* on the development and psychometric evaluation of the HIV stigmatizing attitudes scale in Tanzania. The measure is separated into two components with strong reliability and validity: moral judgment and interpersonal distance. ^37^

According to the examination of the psychometric properties of nine HIV preventive behavior scales, there are nine HIV preventive behavior scales. It was determined to be content valid, structurally valid with discrimination, and to have adequate reliability (Cronbach’s reliability coefficient = .77), which may explain the group homogeneity of the data in the risk condition of 6 months, resulting in consistent measurement. ^27^ In addition, it was six items more confident than the PrEP self-efficacy measure (Cronbach’s alpha coefficient = .62) examined by Mueses-Marn *et al.*, 2021 ^12^ are just as reliable as the original measurement (Cronbach’s alpha = .77). ^13^ Considering the convergent and discriminant validity of the HIV Preventive Behavior Scale, the nine items can be viewed as evidence of the structural validity of the scale. The HIV Preventive Behavior Scale correlates with the HIV Preventive Behavior Scale and can also be examined in terms of self-protective behaviors during sexual encounters. ^13^ It can be shown that this study is comparable to the score on the Perceived Risk of HIV Scale, which is positively related to the score on the Risk Behavior Assessment ( *r*_xy_ = .63, *p* ≤ .001). ^9^ In addition, there was no correlation between the 9-item HIV Preventive Behavior Scale and the Thai language learning attitude scale. This is because the HIV-preventive behavior measurement content is unrelated to the perception of learning Thai. This demonstrates the instrument’s discriminant validity.

### Limitations and implications

The primary limitation of this study was the questionnaire response rate of the sample. This research was conducted using an online survey, which made it difficult to distinguish between the MSM group and those with risk behaviors for HIV infection during the prior six months. This may result in erroneous study results. Next, question number 4, the use of condoms when having sex, this topic was eliminated from the EFA analysis as component 3, but the researcher explored integrating it with component 2, which relates to HIV-preventative behaviors. Since the content of the question was primarily evaluated, it was determined that the content was compatible with the question in the element of group component 2 without starting with the EFA analysis results. ^39^ However, the CFA analysis revealed that the measurement models were coherent when Question 4 and Component 2 were joined.

## Conclusions

The HIV Preventive Behavior Scale was discovered to have two components: rejecting and avoiding the risk of HIV infection and preventative behaviors before and during sexual activity. The measurement model was well consistent with the empirical data when assessing the CFA from nine items. In this regard, the full version of the exam has a specific amount of confidence that it is acceptable. However, further research is required on convergent and discriminant validity, as the correlation between comparable and dissimilar measures remains poor, as well as on the components of evaluating HIV prevention behavior in men. This is because many studies failed to differentiate between denial and avoidance behaviors at risk of HIV infection and protective behaviors before and during sex. ^38^

## Data Availability

Figshare: Raw data of psychometric properties’ HIV Preventive Behavior Scale among Thai MSM, https://doi.org/10.6084/m9.figshare.22491319.v6. ^40^ This project contains the following underlying data:
1. CFA 9 item.docx (CFA analysis results) 2. EFA.docx (EFA analysis results) 3. Reli_corre.docx (analysis results of reliability, convergent and discriminant validity) 4. Raw data.sav (raw data file) 5. Questionnaire.docx (questionnaire file and google form: https://docs.google.com/forms/d/e/1FAIpQLSfzsgYqrNlWYVwUCOkRuRDRGjHipVFHQ6Hf44IBUvOKKPqiww/viewform?usp=sf_link) Data are available under the terms of the Creative Commons Attribution 4.0 International license (CC BY 4.0).
