# HIV preventive behavior scale for Thai men who have sex with men (MSM): development and psychometric properties

**DOI:** 10.12688/f1000research.133299.2

**Published:** 2026-03-18

**Authors:** Passakorn Koomsiri, Nanchatsan Sakunpong

**Affiliations:** 1 Behavioral Science Research Institute, Srinakharinwirot University, Bangkok, 10110, Thailand

**Keywords:** HIV, MSM, Preventive Behavior, Psychometric Properties, Scale

## Abstract

**Background** In foreign countries, many scales have been developed to measure HIV prevention behavior. However, there are only a few developed scales among MSM in Thailand and they are not up to date. The objective of this study is to examine the reliability and validity of the HIV preventive behavior measure among Thai men who have sex with men (MSM).

**Methods** The sample comprised 424 Thai MSM aged 25 years or older who had at least one sexual encounter in the past six months. The total sample was randomly divided into two equal subsamples: one used for exploratory factor analysis (EFA) to identify underlying structures, and the other for confirmatory factor analysis (CFA) to verify model fit. Cronbach’s alpha was employed as the primary reliability coefficient because it reflects internal consistency, and the data collection was conducted only once. Convergent and discriminant validity were examined through Pearson’s correlation coefficients with theoretically related and unrelated constructs to assess coherence and distinctiveness of the measure.

**Results** The measure comprised nine items forming two components: (1) denial and avoidance of HIV risk, and (2) self-protective actions before and during sexual activity. CFA indicated an excellent model fit (χ ^2^ = 36.56, *p* = .06, χ ^2^/df = 1.46, GFI = 0.96, CFI = 0.98, RMSEA = 0.05), meeting recommended criteria for a valid psychometric model. Internal consistency was acceptable (Cronbach’s α = .77). Significant correlations with related scales (he AIDS risk behavior avoidance scale and the AIDS prevention scale) ( *r* = .21 and.16, *p* < .01) and the absence of correlation with an unrelated measure (Thai Learning Attitude Scale) supported convergent and discriminant validity.

**Conclusions** The scale demonstrated sound psychometric properties and is applicable for future interventions to promote HIV preventive behaviors among Thai MSM.

## Introduction

Men who have sex with men (MSM) constitute a sexual-minority group that is exposed to stigma and discrimination, which can adversely affect health. The Minority Stress Model shows that sexual-minority individuals experience stress arising from distal stressors (e.g., prejudice and discrimination) and proximal processes (e.g., internalized homophobia and concealment), which, in turn, elevate risk for mental and physical health problems, including HIV-related vulnerabilities. ^1^ ^,^ ^2^ In Thailand, recent reports underscore the magnitude of the problem: sexually transmitted infections were found in 7.2% to 21.4% of MSM samples between 2014–2018. ^3^ This epidemiologic patterns highlight the continuing need to understand and strengthen HIV prevention behaviors in Thai MSM.

HIV infection, if untreated, progressively depletes CD4 cells and leads to opportunistic infections and AIDS; while early treatment markedly improves outcomes, prevention remains essential for both people living with HIV and those at risk. ^4^ ^,^ ^5^ In this study, HIV prevention behavior refers to actions undertaken to reduce acquisition and transmission risk (e.g., safer sex practices, regular testing, and uptake/adherence to biomedical prevention). Conceptually, prevention behavior can be framed by health-behavior theories. The Health Belief Model (HBM) emphasizes perceived susceptibility and severity, perceived benefits and barriers, and self-efficacy as drivers of preventive action, while the Theory of Reasoned Action (TRA) links attitudes and subjective norms to behavioral intentions. ^6^ Self-efficacy is another key psychological factor influencing HIV prevention behaviors; people who believe in their ability to protect themselves are more likely to practice safer sex and seek testing. ^7^ These frameworks align with current HIV prevention options (e.g., PrEP and PEP) and underscore psychological determinants of consistent preventive behavior. ^8^ ^,^ ^9^

Measurement of HIV prevention behavior has advanced internationally, with multiple self-report scales demonstrating psychometric support (e.g., Perceived Risk of HIV Scale and instruments assessing knowledge, motivation, and self-efficacy for PrEP). ^10^ ^–^ ^12^ However, in Thailand, being used scales for MSM, such as the AIDS Risk Behavior Avoidance Scale and the AIDS Preventive Behavior Scale, were developed over a decade ago, ^13^ ^,^ ^14^ preceding major shifts in the prevention landscape (e.g., promoting sexual well-being, changes in social contexts and service delivery). ^8^ ^,^ ^9^ This temporal gap raises concerns about content relevance and construct alignment for contemporary Thai MSM.

Accordingly, we developed an updated HIV preventive behavior scale tailored to Thai MSM and evaluated its psychometric soundness within the Thai sociocultural context (including cognitive interviewing for content relevance). Specifically, we examined reliability and construct validity and tested convergent and discriminant validity against theoretically related and unrelated constructs. Our goal is to provide a brief, theory-informed, and context-appropriate measure that can inform surveillance, research, and the design and evaluation of future HIV prevention interventions for Thai MSM.

## Methods

This cross-sectional study examined the psychometric properties of the HIV preventive behavior scale among MSM. A total of 424 participants completed an online questionnaire between August and November 2022. A cross-sectional, single administration is sufficient for initial validation to evaluate internal consistency, dimensionality (EFA/CFA), and convergent/discriminant validity, consistent with established guidance for scale development. ^15^ Ethical approval was granted by the Srinakharinwirot University Human Research Ethics Committee (SWUEC-G-512/2564E; January 19, 2022).

### Sampling

Snowball sampling was employed to access MSM who are difficult to reach via probability frames and to trusted peer networks for online recruitment about HIV-preventive behaviors. This approach is commonly used in studies of stigmatized or hard-to-reach populations due to feasibility and rapid data collection. Recognized limitations include selection bias, over-representation of homogeneous peer clusters, and unknown sampling probabilities. To mitigate these concerns, multiple heterogeneous “seeds” were initiated across venues and communities, namely (1) the Thai Red Cross AIDS Research Centre; (2) the Institute of HIV Research and Innovation (IHRI); (3) Community-Based Treatment (CBTx) and the Community-based Harm Reduction and Rehabilitation Service at Public Health Service Center 41 Khlong Toei, Bangkok Metropolitan Administration; (4) the Rainbow Sky Association of Thailand; and (5) the Mplus Foundation, Chiang Mai. Standardized eligibility screening was applied, referrals per participant were capped, and sample composition was monitored throughout data collection to minimize dominance of any single network.

Eligibility was limited to MSM aged ≥25 to target working-age adults in Thailand with independent income and greater autonomy in health decisions, which shape HIV-preventive behaviors and service access differently from younger student populations. This enhances relevance to community/clinical use. The subjects identified themselves as MSM at least once within the previous six months, and were willing to answer the online HIV preventive behavior scale using a Google form. A total of N = 424 respondents was recruited and randomly split into two independent subsamples for factor analysis: Exploratory factor analysis (n = 212) and Confirmatory factor analysis (n = 212). An item-to-sample ratio of approximately 20:1 was used for initial validation. With 9 items of HIV preventive behavior scale, this yields a minimum of 180 participants per analysis, exceeding this benchmark and aligning with guidance that typically recommends 10–20 participants per item for factor models and absolute N ≥ 200 for stable factor recovery and fit evaluation. ^16^ ^,^ ^17^

### Instruments

*HIV preventive behavior scale*. A brief, context-appropriate scale was developed for Thai MSM to capture contemporary prevention practices not fully reflected in adult Thai measures. Items were adapted from the instruments of Pimthong and Bhanthumnavin ^13^ ^,^ ^14^ and refined through cognitive interviewing ^18^ with three experts who each had ≥5 years of experience in HIV prevention among Thai MSM. Each expert completed two interview sessions (60–90 minutes per session). Interviews assessed item comprehension, appropriateness for Thai MSM, and alignment with HIV-prevention behaviors, using think-aloud and probing techniques to identify ambiguous or double-barreled wording; revisions prioritized clear Thai phrasing and domain alignment. Qualitative analysis of the cognitive-interview data yielded three thematic categories (reported in the Results), which informed an initial 34-item pool mapped to key HIV-prevention behaviors. Content validity was quantified using the Index of Item Congruence (IOC) ^19^ based on ratings from another three independent experts. Items with IOC < .50 were removed or revised, and those with corrected item–total correlations < .30 were further refined to enhance content relevance and internal coherence prior to field testing. The final instrument comprised 9 items loading on two components: *risk avoidance for HIV infection* and *self-protection behavior before and during sexual activity*. All items are positively keyed on a five-point frequency scale (1 = never to 5 = routinely), referencing behaviors over the preceding six months, with higher total scores indicating higher HIV-preventive behavior. The construct is defined as self-protective actions enacted before and during sexual activity, together with effective regulation of sexual emotions, to reduce acquisition and transmission risk.

*AIDS risk behavior avoidance scale*, developed by Pimthong in 2011, ^13^ ^,^ ^14^ it measures behavioral intent or a person’s readiness to attempt to avoid actions or activities that lead to AIDS risk. By choosing to act or not to act, such as changing sexual partners often taking drugs or intoxicants watching porn, and choosing places that should or should not go, such as entertainment venues, and gay saunas, a total of 12 items, with 7 positive items and 5 negative items. The responses are rated on a 6-point scale ranging from the truest to not true at all. Scores were calculated using a total score of 12–72. If the score was high, it indicated that there was a high risk of AIDS avoidance behavior. For the psychometric characteristics, the discriminant power ranged from 4.74 to 8.93, the item correlation coefficient with the total score was .32 to.64, and the reliability for the whole version with the alpha coefficient was.83. with empirical data with a χ ^2^ = 34.70, df = 46, *p*-value = .89, NFI = .94, GFI = .95, AGFI = .91, SRMR = .049, CFI = 1.00, and RMSEA = 0.0. This scale was used to examine convergent validity with the HIV prevention behavior scale.

*AIDS prevention scale*, developed by Pimthong in 2011, ^13^ ^,^ ^14^ it is a model to measure a person’s sexual behavior. By considering the method of sexual intercourse, such as using a condom with lubricant every time you have anal sex. Not swallowing partner’s semen, etc. There were a total of 10 items, 4 positive and 6 negative items. Each item had a 6-point evaluation scale ranging from very true to absolutely false by measuring 3 behavioral components. The aspects related to the risk of HIV infection during sex were 1) no risk, 2) moderate risk, and 3) high risk. The score is calculated using a total score of 10–60. If the score is high, it indicates that AIDS prevention behaviors while having sex are high. For the psychometric characteristics, the discriminant power ranged from 4.09 to 9.90, the item correlation coefficient with the total score was .20 to.62, and the reliability for the whole version with the alpha coefficient was.77. with empirical data with a χ ^2^ = 23.52, df = 30, *p*-value = .70, NFI = .93, GFI = .96, AGFI = .92, SRMR = .049, CFI = 1.00, and RMSEA = 0.0. This scale was used to examine convergent validity with the HIV prevention behavior scale.

*Thai Language Learning Attitude Scale*, developed by Samrongthong in 2011 ^20^ as a model to measure the attitude towards learning the Thai language. There are a total of 20 items. Answers are self-exploratory. Questions are both positive and negative items. Then choose a response from 5 levels: strongly disagree, disagree, not sure, agree, and strongly agree. There was reliability from internal consistency analysis with Cronbach’s reliability coefficient of. 85. This scale was used to examine discriminant validity with the HIV prevention behavior scale.

### Data analysis

*Qualitative data analysis*

Transcripts were de-identified and content-coded by item–probe segments. Codes were iteratively grouped and synthesized into categories with brief verbatim excerpts as evidence. Categories were summarized in an item-by-category matrix to guide revision, cut-off decisions (rewording, examples, response options) or generate new items.

*Quantitative data analysis*

After developing the draft HIV preventive behavior scale from cognitive interviewing, we first examined corrected item–total correlations (target ≥ 0.30) and internal consistency via Cronbach’s alpha (acceptable ≥ 0.70). Construct validity was assessed in two stages. For the EFA, we used principal axis factoring with varimax rotation; factorability was verified (KMO ≥ 0.70; Bartlett’s p < .001). ^16^ Items were retained if primary loadings were ≥ 0.30; items with evidence of redundancy/multicollinearity (inter-item r > 0.80) were removed. For the CFA, model fit was evaluated against conventional thresholds (χ ^2^/df < 3.0, CFI ≥ 0.90, TLI ≥ 0.90, RMSEA ≤ 0.08, SRMR ≤ 0.08). ^21^ To examine convergent/discriminant patterns, we computed Pearson correlations with theoretically related measures (AIDS Risk Behavior Avoidance Scale, AIDS Preventive Behavior Scale) and an unrelated construct (Thai Language Learning Attitude Scale), expecting significant correlation with the former and no significant correlation with the latter.

### Procedures and consent

Data were collected via a secure google form which need to use password to log-in. The landing page of the form presented an information sheet describing the study purpose, procedures, inclusion criteria (≥25 years), expected time, potential risks/benefits, voluntary nature of participation, and contacts for questions or concerns. Participants provided explicit electronic consent by ticking an “I agree to participate” box before any survey items were shown; those who did not consent were automatically exited from the survey. Participants could skip any item and withdraw at any time prior to submission without penalty.

To maintain anonymity, the survey did not request names, phone numbers, or other direct identifiers. IP addresses and device metadata were not retained in the analytic dataset. Responses were stored under randomly generated codes and exported in de-identified form. To ensure confidentiality, data were kept on encrypted, password-protected drives with access limited to the principal investigator and authorized research staff; reporting used only aggregated results and non-identifiable quotes. Data handling adhered to the Institutional Review Board (IRB) requirements and applicable national regulations. The procedures complied with the Declaration of Helsinki and guidance for research involving vulnerable populations. For the cognitive interviews with experts, participants received a formal request letter via their affiliations along with an information sheet. Written (or recorded verbal) consent was obtained before the session; permission to audio-record was sought separately. Experts could decline or stop the interview at any point, and any illustrative quotations were anonymized in dissemination.

## Results

### Qualitative item refinement and domain structure

Cognitive interviewing yielded three categories of HIV preventive behaviors among MSM:

1. Denial and avoidance of HIV risk; behaviors that minimize or steer away from situations with elevated exposure (e.g., downplaying susceptibility, ignoring risk cues, avoiding venues/partners/substances linked to condomless sex or chemsex.
*“Outdoor activities should be avoided because they can increase sexual risk”* (Participant 1)
*“Avoiding transient partnerships helps minimize risk, frequent partner changes markedly increase the likelihood of infection.”* (Participant 2)
*“When friends start mixing drugs at parties, I need to head home before it gets risky.”* (Participant 1)
2. Self-protective actions before and during sexual activity; concrete risk-reduction practices (e.g., PrEP/PEP uptake and adherence, consistent condom and lubricant use, STI testing before new partners, discussing HIV/PrEP status and safer-sex preferences, negotiated safety).
*“Condoms plus lubricant remain foundational; they prevent breakage and increases consistency..”* (Participant 1)
*“Before a new partner, STI/HIV testing and discussing recent results set a safety baseline for both parties.”* (Participant 3)
3. Appropriate sexual response; communication and regulation of sexual needs, emotions, and boundaries that protect one’s own and partners’ wellbeing (e.g., stating consent and limits, refusing unwanted practices, seeking mutual pleasure without pressure, and drawing on self-care such as mindfulness/spirituality/self-acceptance to support safer choices).
  “ *… Less focus on sexual cues and more on other life pleasures; having a mental anchor may reduce sexual preoccupation.”* (Participant 1, follow-up)
*“Activities like aerobic dance, exercise, and running can reduce sexual drive and help people stay safe in love with acceptable arousal.”* (Participant 2)

The categories that emerged from the cognitive interviews were generated through an iterative and systematic analytic process. First, all interview data were transcribed verbatim and reviewed multiple times to achieve familiarization with participants’ interpretations and response processes. Meaning units related to how participants understood, interpreted, and responded to each item were then identified and coded. Next, similar codes were grouped together to identify recurring patterns in participants’ comprehension, perceived relevance, and suggested revisions. Through constant comparison across cases, these patterns were refined into broader conceptual categories. The research team engaged in discussion to reach consensus on the boundaries and definitions of each category, ensuring that the categories accurately reflected participants’ cognitive processes and were grounded in the data. These categories subsequently informed item refinement and conceptual clarification of the measure.

Quotations under denial and avoidance of HIV risk demonstrate how participants appraise and minimize exposure (e.g., avoiding high-risk venues/contexts, recognizing partner turnover and substance-linked encounters), thereby justifying items in the risk-avoidance component. Quotations under self-protective actions before and during sexual activity specify concrete practices (e.g., condom negotiation/use, testing routines, and avoiding intoxicated sex to preserve adherence), directly informing items in the self-protection component. Quotations related to appropriate sexual response clarify mechanisms of boundary-setting and emotion regulation that support consistent prevention and sexual well-being.

### Sample characteristics and item screening

The psychometric sample comprised 424 MSM reporting sex with a man in the past six months (mean age = 32.08, SD = 6.42). Content validity, assessed by three expert reviewers, showed item–objective congruence (IOC) ranging 0.66–1.00 across 19 items. Items were then screened via corrected item–total correlations (CITC) using a retention threshold of ≥0.30. Only nine retained items exhibited CITC values 0.33–0.47. Assumptions of univariate normality were acceptable (skewness and kurtosis within ±2), supporting subsequent factor analyses.

### Construct validity

Pairwise associations among the nine items (N = 424) were examined using Pearson correlations; 35 of 36 pairs were significant at *p* < .05 (range *r* = .11–.58; no pair exceeded.90), with one nonsignificant pair (items 2 and 4). Sampling adequacy was acceptable (KMO = .78) and Bartlett’s test of sphericity was significant (χ ^2^(36) = 843.73, *p* < .001), supporting factor analysis. An exploratory analysis was conducted on a random half-sample (n = 212) using PCA with varimax rotation. Component retention followed multiple criteria: eigenvalue >1, inspection of the scree plot for an elbow, simple structure/interpretability, and consistency with the a priori content framework. Items were retained on a component when primary loadings ≥.40 and cross-loadings < .30 ^16^ ^,^ ^21^ items failing these thresholds were reviewed for content coherence and communality.

The initial solution yielded three components with eigenvalues >1. Component 1 (denial/avoidance of HIV risk) showed loadings .75–.83 for its three items. Component 2 (self-protective actions before/during sexual activity) showed loadings .46–.73 for items 5–9. Item 4 emerged as a singleton on Component 3, with insufficient item count for a stable factor and content overlap with self-protective actions. Given its acceptable conceptual alignment (pre-sex planning/safer-sex enactment), item 4 was reassigned to Component 2, yielding a two-component structure consistent with the theoretical model: (1) denial/avoidance of HIV risk and (2) self-protection before/during sexual activity. Details are in Table 1.

**Table 1.** Factor loading, Eigenvalues, % of Variance, and Cumulative % (n=212).

Items	Component 1	Component 2	Component 3
Item 1 You avoid having sexual contact. When it is discovered that your partner is not protected by wearing a condom. คุณหลีกเลี่ยงที่จะมีเพศสัมพันธ์ เมื่อพบว่าคู่นอนของตนไม่ป้องกันด้วยการสวมถุงยางอนามัย	.75		
Item 2 You decline when a buddy invites you to a potentially risky sex activity, such as group sex or traveling to a meeting spot for sex. Substance Dependency. คุณปฏิเสธเมื่อเพื่อนชวนไปร่วมกิจกรรมที่อาจมีความเสี่ยงต่อการมีเพศสัมพันธ์ที่ไม่ปลอดภัย เช่น เซ็กส์หมู่ การไปแหล่งนัดพบเพื่อมีเพศสัมพันธ์ การใช้สารเสพติด	.83		
Item 3 You consistently refuse to switch partners. คุณปฏิเสธการเปลี่ยนคู่บ่อย ๆ	.76		
Item 4 You use a condom during sexual activity. คุณใช้ถุงยางอนามัยเมื่อมีเพศสัมพันธ์			.89
Item 5 You use oil-free lubricants during sexual activity. คุณใช้สารหล่อลื่นที่ไม่มีส่วนผสมของน้ำมันในขณะมีเพศสัมพันธ์		.46	
Item 6 You avoid touching the wound immediately after sexual activity. คุณพยายามไม่สัมผัสบาดแผลโดยตรงหลังจากมีเพศสัมพันธ์		.54	
Item 7 When you need to, you can show your sexual feelings in many ways, such as masturbation, spiritual activities, and recreational activities. เมื่อมีความต้องการ คุณระบายความรู้สึกทางเพศด้วยวิธีการต่าง ๆ ได้ เช่น การสำเร็จความใคร่ด้วยตนเอง กิจกรรมเสริมสร้างจิตวิญญาณ กิจกรรมนันทนาการ		.59	
Item 8 You engage in sexual action while avoiding being harmed or producing blood or lymph. คุณมีเพศสัมพันธ์โดยพยายามไม่ทำให้เกิดบาลแผล เลือด น้ำเหลือง		.66	
Item 9 You engage in sexual activity without causing physical harm to your partner. คุณมีเพศสัมพันธ์โดยไม่ใช้ความรุนแรงด้านร่างกายที่จะทำให้เ กิดบาดแผลกับคู่นอน		.73	
Eigenvalues	2.61	1.49	1.01
% of Variance	29.01	16.60	11.30
Cumulative %	29.01	45.60	56.90

The two-component structure was tested in the another half-sample (n = 212) using CFA and showed adequate global fit, meeting or exceeding conventional benchmarks: nonsignificant χ ^2^ (χ ^2^ = 36.56, p = .06), χ ^2^/df = 1.46 (≤3.0), GFI = .96 (≥.90), CFI = .98 (≥.95), TLI = .96 (≥.95), RMSEA = .05 (≤.06), and RMR = .07 (≈ ≤ .08), ^17^ supporting the adequacy of the model. At the item level, Component 1: denial and avoidance of HIV risk comprised items 1–3 with standardized loadings (β) = .60–.85, SE = .15–.20, *t* = 7.32–7.55, *p* < .01, and R ^2^ = .36–.73. Component 2: self-protective actions before and during sexual activity comprised items 4–9 with β = .41–.74, SE = .17–.26, *t* = 4.15–6.29, *p* < .01, and R ^2^ = .13–.54. The latent correlation between the two components was.58, indicating related but distinct constructs ( Table 2). Together, the fit indices and item-level criteria justify the allocation of items to the two reported components and the reassignment of item 4 based on statistical thresholds and construct coherence. ^1^

**Table 2.** Factor loading, Standard Error, t Values, Coefficient of Determination by CFA (n = 212).

Items	*b*	*SE*	*t*	*R* ^2^
**Dimension 1, Denying and avoiding the risk of contracting HIV (F1)**				
Item 1 You avoid having sexual contact. When it is discovered that your partner is not protected by wearing a condom. (X1)	.60	-	-	.36
Item 2 You decline when a buddy invites you to a potentially risky sex activity, such as group sex or traveling to a meeting spot for sex. Substance Dependency. (X2)	.85	.20	7.55 **	.73
Item 3 You consistently refuse to switch partners. (X3)	.66	.15	7.32 **	.44
**Dimension 2, Self-protective behavior before and during sex (F2)**				
Item 4 You use a condom during sexual activity. (X4)	.49	-	-	.24
Item 5 You use oil-free lubricants during sexual activity. (X5)	.37	.21	4.15 **	.13
Item 6 You avoid touching the wound immediately after sexual activity. (X6)	.74	.26	6.29 **	.54
Item 7 When you need to, you can show your sexual feelings in many ways, such as masturbation, spiritual activities, and recreational activities. (X7)	.41	.17	4.51 **	.17
Item 8 You engage in sexual action while avoiding being harmed or producing blood or lymph. (X8)	.72	.26	6.26 **	.52
Item 9 You engage in sexual activity without causing physical harm to your partner. (X9)	.63	.26	5.88 **	.39

** *p* < .01.

Consistent with empirical data, the following two components of the HIV Preventive Behavior scale model were identified by CFA: Chi-square: χ ^2^ = 36.56, *p *= .06, Relative Chi-square: χ ^2^/df = 1.46, Goodness of Fit Index: GFI = .9, 6, Comparative Fit Index: CFI = .8, Adjusted goodness of fit index: AGFI = .94, Root Mean Square Residual: RMR = .07, Root Mean Square Error of Approximation: RMSEA = .05, Root mean square residual: TLI = .96. Overall, the model matches the empirical data well (good fit).

### Reliability, convergent, and discriminant validity

Internal consistency from Cronbach’s reliability coefficient was used to determine that the 9-item HIV Preventive Behavior Scale’s reliability was equivalent to .77 Pearson product-moment correlation coefficients, which were used to examine convergent and discriminant validity among 424 individuals Thai MSM. There are positive correlations between the HIV Preventive Behavior Scale (SUMPHIV) with the AIDS risk behavior avoidance scale (SUML), and the AIDS prevention scale (SUMA) were statistically significant at the .01 level, with correlations of .21 and .26, respectively. There was no correlation between the HIV Preventive Behavior Scale and the Thai Language Learning Attitude Scale (SUMT) (see Table 3).

**Table 3.** Relationship between HIV Preventive Behavior Scale and AIDS Risk Scale, AIDS prevention measure, and attitude measure toward learning the Thai language (n = 424).

Scales	SUMPHIV	SUML	SUMA	SUMT
**SUMPHIV**	-	21 **	.26 **	.08
**SUML**		-	.25 **	.16 **
**SUMA**			-	.18 **
**SUMT**				-
Mean	32.21	43.40	36.44	70.65
Standard deviation	6.39	7.09	6.28	17.43

** *p* < .01.

## Discussion

This study advances Thai measurement work on HIV prevention by updating content and structure beyond earlier instruments by Pimthong and Bhanthumnavin ^13, 14^ to reflect contemporary practices (adding risk situations and sexual well-being) that were not widely captured when prior tools were developed. In addition, the scale integrates constructs previously assessed by two separate instruments into a single, brief measure, thereby reducing respondent burden and administration time while preserving coverage of both risk-avoidance and self-protective behaviors.

The resulting two-component model (1) denial/avoidance of HIV risk and (2) self-protective actions before and during sexual activity, showed strong fit, convergent and discriminant validity (see Results). The moderate latent correlation between components (.58) indicates related but distinct facets: minimizing exposure opportunities versus enacting concrete protective routines. This structure converges with other two-factor solutions in HIV-related constructs, e.g., HIV risk perception among Hispanic-American youth. ^11^ Relative to earlier Thai measures, ^13, 14^ the present scale foregrounds biomedical-era prevention while preserving the dual emphasis on risk minimization and protective behavior, thereby offering a concise, behavior-proximal assessment aligned with current service landscapes.

The two components are theoretically coherent within established health-behavior frameworks. Denial/avoidance corresponds to Health Belief Model (HBM) constructs of perceived susceptibility/severity and barriers (e.g., steering clear of venues/partners/substances linked to condomless sex), and is also consistent with Theory of Reasoned Action/Planned Behavior pathways from attitudes, norms, and perceived control to intentions and behavior (e.g., condom and lubricant negotiation). ^6^ ^,^ ^7^ The emphasis on emotion/impulse regulation during sexual decision-making is compatible with self-regulatory and dual-process perspectives, ^7^ linking momentary control to sustained prevention. Taken together, the model can be situated within the Information–Motivation–Behavioral Skills (IMB) framework ^22^: denial/avoidance indexes risk appraisal and environmental management, while self-protection indexes motivation/skills for enactment.

Cultural context in Thailand likely shapes both dimensions. Social norms around masculinity, discretion, and HIV-related stigma may encourage denial or avoidance (e.g., downplaying risk when a guy appears good-looking and healthy; avoiding settings associated with chemsex or condomless encounters.). At the same time, self-protective actions are facilitated or constrained by interpersonal scripts such as kreng-jai (reluctance to impose in Thai culture) that can complicate condom/lubricant negotiation. These sociocultural levers clarify why both components are necessary: avoidance strategies reduce exposure opportunities, whereas intentional protective actions translate prevention goals into everyday sexual contexts.

Psychometrically, internal consistency (α = .77) and convergent/discriminant patterns support initial reliability and structural validity in Thai MSM. This suggests structural generalizability, while differences in stigma intensity, service systems, and sexual-script norms may underscore the importance of context-sensitive implementation.

A practical implication is to embed the two-component model directly into program design and policy for MSM. First, use the 9-item scale as a brief screening tool in community clinics, and online outreach to segment clients: higher denial/avoidance scores trigger modules on risk-cue literacy; higher self-protection gaps trigger skills-based supports (low-conflict condom scripts compatible with *kreng-jai*, pre-sex planning checklists, communication “micro-scripts,”. Second, integrate both components into IMB-consistent packages, ^22^ information (myth-busting, visual risk cues), motivation (normative messaging from MSM peers), and behavioral skills (condom/lube demonstrations, brief negotiation practice) to initiate HIV preventive intervention. Third, operationalize delivery through peer navigators and digital tools (chat/app prompts before high-risk time windows). Finally, align monitoring with the scale: set component-specific thresholds to trigger referrals, track pre–post change for each subscore, and include indicators (e.g., PrEP uptake/adherence, timely testing) in routine dashboards to inform continuous quality improvement and resource allocation.

### Limitations

Several limitations warrant consideration. First, recruitment via an online, snowball-based survey introduces selection bias (internet access, network homophily) and unknown sampling probabilities, which can limit generalizability beyond MSM engaged with digital/community networks. Second, reliance on self-reported behaviors over a six-month window is vulnerable to recall error and social desirability, potentially inflating protective reports (e.g., condom use) and underreporting risk contexts (e.g., chemsex). Third, the sample’s age composition (≥25 years) and sexual-orientation profile may yield sample homogeneity, constraining applicability to younger MSM and other subgroups (e.g., bisexual-identified men, gender-diverse partners). Fourth, the cross-sectional design precludes test–retest reliability, temporal stability, and predictive validity (e.g., incident STI/HIV).

Future research should broaden sampling frames (e.g., respondent-driven or venue-based strategies, multiple regions) and include younger MSM to assess age-related differences. Methodological refinements include test–retest assessment, longitudinal designs for predictive validity, incorporation of social desirability scales, ecological momentary assessment to reduce recall bias, and triangulation with clinic/biomarker data (testing history, PrEP dispensing, STI diagnoses). Cross-cultural studies should examine how sexuality norms, masculinity scripts, stigma, and service access shape the two domains in other Thai regions and countries. Finally, intervention trials can use the 9-item scale to tailor content, addressing denial/avoidance (risk-cue literacy, venue/substance harm-reduction) and enhancing self-protection (condom negotiation, PrEP/PEP adherence), and to track component-specific change as part of routine program monitoring.

## Conclusions

The 9-item HIV Preventive Behavior Scale developed for Thai MSM demonstrates sound psychometric performance and a parsimonious two-component structure; denial/avoidance of HIV risk and self-protective actions before and during sexual activity, supported by satisfactory model fit, acceptable internal consistency (α = .77), and coherent convergent/discriminant patterns. By integrating constructs previously assessed across separate Thai instruments into a single brief measure, the scale reduces respondent burden while preserving coverage of contemporary prevention practices (e.g., condom/lubricant use, risk-context management). The tool is suited for screening, program tailoring, and monitoring within community and clinical settings, and its theory-aligned domains map readily onto IMB/HBM and related behavioral frameworks to guide intervention content. Acknowledging limitations of online snowball sampling and self-report, further work should examine measurement invariance, test–retest and predictive validity, and applicability to younger MSM and diverse regional contexts. Overall, the scale offers a concise, culturally grounded metric to inform HIV prevention policy and practice for MSM in Thailand.

## Data Availability

Figshare: Raw data of psychometric properties’ HIV Preventive Behavior Scale among Thai MSM, https://doi.org/10.6084/m9.figshare.22491319.v6. ^23^ This project contains the following underlying data:
1. CFA 9 item.docx (CFA analysis results) 2. EFA.docx (EFA analysis results) 3. Reli_corre.docx (analysis results of reliability, convergent and discriminant validity) 4. Raw data.sav (raw data file) 5. Questionnaire.docx (questionnaire file and google form: https://docs.google.com/forms/d/e/1FAIpQLSfzsgYqrNlWYVwUCOkRuRDRGjHipVFHQ6Hf44IBUvOKKPqiww/viewform?usp=sf_link) Data are available under the terms of the Creative Commons Attribution 4.0 International license (CC BY 4.0).
