# Supplementary material for: HIV preventive behavior scale for Thai men who have sex with men (MSM): development and psychometric properties
Source: F1000Res. 2026 Mar 18;12:513. Originally published 2023 May 18. [Version 2] doi: 10.12688/f1000research.133299.2 (PMC13090760; doi:10.12688/f1000research.133299.2)
Supplement: Peer review history

# Reviewer report: Reviewer response for version 2

**DOI:** 10.5256/f1000research.197233.r468631

**Published:** 2026-04-17

**Authors:** Roghieh Nooripour

**Affiliations:** 1 Alzahra University, Tehran, Tehran Province, Iran

After careful review, I confirm that the authors have addressed all previously raised concerns in a thorough and scientifically appropriate manner. The revisions have improved clarity, methodological transparency, and consistency across sections, particularly in the alignment between results and conclusions. No major issues remain that would compromise the scientific soundness of the study. The manuscript now meets the standards for indexing. I appreciate the authors’ careful and responsive revisions, which have strengthened the overall quality and credibility of the work.

Is the work clearly and accurately presented and does it cite the current literature?

No

If applicable, is the statistical analysis and its interpretation appropriate?

No

Are all the source data underlying the results available to ensure full reproducibility?

No

Is the study design appropriate and is the work technically sound?

Partly

Are the conclusions drawn adequately supported by the results?

No

Are sufficient details of methods and analysis provided to allow replication by others?

No

Reviewer Expertise:

Counselling psychology; clinical psychology; adolescent mental health; mindfulness-based interventions; cognitive behavioural therapy (CBT); neurofeedback and cognitive rehabilitation; emotion regulation and executive functions; high-risk behaviours and addiction; cyberbullying and digital mental health; psychometrics, scale development, and validation; quantitative and qualitative research methods; longitudinal and intervention study design; psychological well-being and resilience; health psychology (including psycho-oncology and chronic illness populations).

I confirm that I have read this submission and believe that I have an appropriate level of expertise to confirm that it is of an acceptable scientific standard.

# Reviewer report: Reviewer response for version 2

**DOI:** 10.5256/f1000research.197233.r469526

**Published:** 2026-03-26

**Authors:** Qingyu li

**Affiliations:** 1 Vanke School of Public Health, Tsinghua University, Beijing, China

Overall, my judgment is that the manuscript addresses an important topic and has clear public health relevance, but it still has substantive problems in construct definition, content validity, and psychometric interpretation. In its current form, I would lean toward **major revision** rather than acceptance. The paper does present a reasonable first attempt at a Thai MSM-specific prevention behavior scale, based on 424 participants split evenly for EFA and CFA, with acceptable internal consistency and apparently good global CFA fit. However, the final 9-item scale does not fully match the broader construct that the manuscript claims to measure, namely contemporary HIV prevention behavior among Thai MSM.

**General assessment**

 This manuscript addresses an important and timely topic: the development of a brief HIV preventive behavior scale for Thai men who have sex with men. The effort to update older Thai measures is meaningful, especially given changes in HIV prevention, including biomedical strategies and shifts in service delivery. The study also has several strengths, including a clearly defined target population, split-sample EFA/CFA, and reporting of internal consistency and convergent/discriminant validity. The reported CFA indices are favorable, and the authors have made visible efforts to strengthen the revised version.

 That said, I have several major concerns. The most important issue is conceptual: the manuscript defines HIV prevention behavior broadly, including safer sex, regular testing, and uptake/adherence to biomedical prevention such as PrEP and PEP, yet the final 9-item scale is much narrower and is largely centered on partner avoidance, condom/lubricant use, injury avoidance, and regulation of sexual feelings. This creates a mismatch between the stated construct and the actual item content. In addition, the factor-analytic decisions need clearer justification, especially because the initial solution yielded three components and one item was reassigned on conceptual grounds. The validity evidence is also still fairly limited for a new scale that is proposed for intervention and program use.

 For these reasons, I think the manuscript has potential, but it needs substantial revision before it can be considered psycho metrically convincing and fully informative for HIV prevention research and practice.

**Major comments**

 1. Abstract

 The abstract is clearer than a minimal draft, but it still overstates what the study has established. The manuscript reports acceptable internal consistency, a two-factor solution, and modest convergent correlations with related measures. However, the conclusion that the scale demonstrated “sound psychometric properties” and is ready for future interventions feels stronger than the presented evidence. The abstract should more cautiously describe this as an **initial validation** or **preliminary psychometric evaluation**, especially because test-retest reliability, criterion validity, predictive validity, and in variance testing were not performed.

 A second issue is construct clarity. The introduction defines HIV prevention behavior as including regular testing and biomedical prevention uptake/adherence, but the abstract does not alert the reader that the final 9-item scale captures only a subset of this wider domain. That gap should be acknowledged even in the abstract, so the reader does not assume that the measure comprehensively covers contemporary HIV prevention practices.

 2. Introduction

 The introduction identifies a relevant gap, namely that older Thai MSM scales may no longer reflect the current prevention context. That rationale is reasonable. However, the manuscript still does not fully bridge the distance between the broad prevention framework introduced in the text and the narrower behavioral content represented in the final measure. The manuscript explicitly defines HIV prevention behavior to include regular testing and PrEP/PEP-related practices, and it cites literature on PrEP-related constructs, but those elements are not retained in the final scale. This creates a conceptual problem: the paper is framed as a modern HIV prevention behavior measure, but the final scale remains closer to a traditional sexual risk reduction instrument.

 The theoretical framing also needs tightening. The introduction mentions the Minority Stress Model, the Health Belief Model, the Theory of Reasoned Action, and later the Information–Motivation–Behavioral Skills framework. At present, these models feel more appended than integrated. The authors should decide whether the scale is primarily behavioral, psycho-social, or both, and then explain more directly how the proposed dimensions arise from that theory. Otherwise, the introduction risks sounding broader and more theoretically complete than the measure itself supports.

 3. Methods

 The methods section is more detailed than in a minimal scale-development report, but several concerns remain.

 First, the scale-development process still needs stronger transparency. The paper states that an initial 34-item pool was mapped to key HIV prevention behaviors, then reduced using IOC and corrected item-total correlations, with a final 9-item scale. However, the reader is not given enough detail about which domains were dropped and why. This is especially important because the qualitative categories explicitly include PrEP/PEP uptake and adherence, HIV/STI testing, and status discussion, yet none of these are clearly represented in the final item set. A table showing domain-to-item mapping from initial pool to final scale would greatly strengthen content-validity claims.

 Second, the use of cognitive interviewing with only three experts raises a methodological concern. Cognitive interviewing is most often used with members of the target population to examine comprehension, response processes, and cultural relevance. Here, the interviews appear to have been conducted with experts, not end users, which limits the strength of the evidence for target-population comprehensibility. The paper should justify this choice much more explicitly and avoid presenting it as a full substitute for user-based qualitative item development.

 Third, the sampling strategy is understandable for a stigmatized population, and the authors do acknowledge limitations of snowball sampling. Still, the decision to include only participants aged 25 years or older substantially narrows the target population. Younger MSM are a key HIV prevention population in many settings. The authors should therefore avoid general language implying applicability to all Thai MSM, unless they can justify why the construct should operate similarly in younger groups.

 Fourth, there is an inconsistency in factor-analytic terminology. In the methods, the authors state that EFA used principal axis factoring with varimax rotation. In the results, the text refers to PCA with varimax rotation. These are not interchangeable procedures. This inconsistency needs correction, and the authors should clearly report which method was actually used.

 4. Results

 The results section is where the main psychometric concerns become most visible.

 The qualitative section identifies three categories: denial and avoidance of HIV risk, self-protective actions before and during sexual activity, and appropriate sexual response. These categories include modern prevention content such as PrEP/PEP use, testing, and HIV/PrEP status discussion. However, the final 9 items do not visibly preserve this breadth. Instead, the retained items focus on condom use, lubricant use, partner switching, risky invitations, injury/wound avoidance, and sexual feeling regulation. This weakens the argument that the final scale adequately reflects the broader domain identified in the qualitative phase.

 The factor analysis also needs more careful interpretation. The exploratory solution initially yielded **three** components with eigenvalues above 1, and item 4 loaded alone on the third component. The authors then reassigned item 4 to component 2 because it conceptually aligned with self-protective actions. That may be reasonable, but it also means the final two-factor model was not the most direct empirical solution. This should be described more cautiously. The current wording makes the final structure sound more naturally supported by the data than it actually was.

 Some item content also raises concern about construct purity. For example, the item about expressing sexual feelings through masturbation, spirituality, or recreation, and the items about avoiding wounds or not physically harming a partner, may reflect broader sexual self-regulation or interpersonal conduct rather than core HIV prevention behavior. These items may be culturally meaningful, but the authors need to explain more clearly why they belong in a scale presented as an HIV prevention behavior measure, especially when more central contemporary prevention behaviors were not retained.

 Finally, the validity evidence is limited. Cronbach’s alpha is acceptable at 0.77, but the convergent validity coefficients are small to modest, and there is no criterion-related validity, known-groups validity, predictive validity, or test-retest reliability. The paper therefore supports preliminary structural validity, not full psychometric maturity.

 5. Discussion

 The discussion is thoughtful in places, especially where it tries to situate the two factors within health-behavior frameworks and Thai sociocultural context. However, it still overstates the scale’s scope and readiness.

 Most importantly, the discussion claims that the present scale “foregrounds biomedical-era prevention,” yet the final retained items do not convincingly do so. The manuscript itself introduces PrEP, PEP, and regular testing as key parts of HIV prevention behavior, and these were mentioned in the qualitative categories, but they are not reflected in the reported 9-item measure. The authors should either revise their claims downward or explain much more clearly why the absence of these domains does not compromise content validity.

 The discussion would also benefit from stronger comparison with prior literature. The paper states that the new tool updates earlier Thai instruments and combines previously separate domains into one measure, which is useful. But it does not sufficiently explain how the present two-factor structure compares with similar HIV prevention behavior instruments or whether this structure is expected to be culturally specific.

 I also think the practical recommendations are somewhat ahead of the evidence. The paper proposes screening, segmentation, monitoring, and intervention tailoring based on this 9-item scale. Those suggestions are interesting, but without known-groups validity, predictive validity, or threshold validation, they remain hypothetical. The discussion should present these as future possibilities rather than current implementation recommendations.

**Minor comments**

 Abstract

 Please check small wording and numerical consistency in the abstract. In one place the convergent correlations are reported as 0.21 and 0.16, whereas the main text/table later report 0.21 and 0.26. This needs to be harmonized.

 Introduction

 There are still multiple language issues and awkward phrases in the introduction, for example “being used scales for MSM” and “This epidemiologic patterns.” These do not prevent understanding but reduce the professional tone of the paper.

 Methods

 Please provide a flow of item reduction from 34 items to 19 items to 9 items, with reasons for removal at each step. This would make the development process much more transparent.

 Please also clarify whether the scale was developed in Thai and then translated into English for the manuscript, and if so, whether any translation/back-translation procedures were used for reporting item content. The current bilingual presentation is useful, but the language handling process is not fully clear.

 Results

 Please define the factor labels more carefully. “Denial and avoidance of HIV risk” may not be the clearest label, because the retained items seem to reflect deliberate avoidance of risky situations rather than psychological denial in the usual sense. A label such as “risk avoidance” may be more precise.

 Please review all statistical reporting for consistency. The manuscript gives fit indices twice, and one repeated sentence appears to contain typographic errors, including “CFI = .8” and mislabeling TLI as “Root mean square residual.”

 Discussion

 The cultural discussion is promising, especially the mention of stigma, masculinity, discretion, and *kreng-jai*. However, it would be stronger if more directly tied back to specific items rather than discussed at a general level.

 The future research section would be improved by explicitly stating the next psychometric steps: test-retest reliability, known-groups validity, predictive validity against actual prevention outcomes, and invariance across age groups and regions. The current version mentions some of these, but the sequence of priorities could be clearer.

Is the work clearly and accurately presented and does it cite the current literature?

Partly

If applicable, is the statistical analysis and its interpretation appropriate?

Partly

Are all the source data underlying the results available to ensure full reproducibility?

Partly

Is the study design appropriate and is the work technically sound?

Partly

Are the conclusions drawn adequately supported by the results?

Partly

Are sufficient details of methods and analysis provided to allow replication by others?

Partly

Reviewer Expertise:

HIV; PrEP; adherence; digital health; intervention; health behavior

I confirm that I have read this submission and believe that I have an appropriate level of expertise to confirm that it is of an acceptable scientific standard, however I have significant reservations, as outlined above.

# Reviewer report: Reviewer response for version 1

**DOI:** 10.5256/f1000research.146278.r409707

**Published:** 2025-09-10

**Authors:** Rodman E Turpin

**Affiliations:** 1 George Mason University, Fairfax, USA

The authors conducted a psychometric analysis of an HIV prevention behavior scale among Thai MSM. Overall the study addresses an important question, is salient with implications for large-scale public health efforts, and will be a strong contribution to the literature. I have a few recommendations to improve clarity and readability.

 1. Minor point: You could save a lot of word space by using MSM as an acronym after its first mention in the introduction.

 2. In the first paragraph, some additional discussion on some of the literature around homophobia as a stressor among MSM (with citation) will be helpful.

 3. In general, review for grammatical errors throughout (such as the first sentence of the methods, where "examination of" should be "examine".

 4. In the construct validity section, "The researcher used a sample of 424 people" should be removed, since the sample size was mentioned one paragraph earlier. Also, saying you used a sample of people can sound exploitative, it's generally better to say you used data rather than a sample.

 5. Future research recommendations should be elaborated on more.

Is the work clearly and accurately presented and does it cite the current literature?

Yes

If applicable, is the statistical analysis and its interpretation appropriate?

Yes

Are all the source data underlying the results available to ensure full reproducibility?

Yes

Is the study design appropriate and is the work technically sound?

Yes

Are the conclusions drawn adequately supported by the results?

Yes

Are sufficient details of methods and analysis provided to allow replication by others?

Yes

Reviewer Expertise:

HIV, STI, racial/ethnic and sexual minorities, latent variable modeling

I confirm that I have read this submission and believe that I have an appropriate level of expertise to confirm that it is of an acceptable scientific standard.

# Reviewer report: Reviewer response for version 1

**DOI:** 10.5256/f1000research.146278.r353209

**Published:** 2024-12-30

**Authors:** Roghieh Nooripour

**Affiliations:** 1 Alzahra University, Tehran, Tehran Province, Iran

**Abstract**

1. The objective stated in the abstract should be more precise. Instead of stating that the study “investigates the psychometric features,” specify the particular psychometric features you aim to assess (e.g., reliability, construct validity, etc.). This will provide the reader with a clearer understanding of the study's purpose.
2. The description of the methods is very brief. Mention how the two-part division of the sample (for exploratory and confirmatory factor analysis) is executed, and clarify the rationale for using Cronbach’s alpha as the primary reliability measure. Also, explain the choice of convergent and discriminant validity tests, and how they are relevant to the study's goals.
3. In the results section, the statistical values (e.g., χ2, p-values, CFI) should be explained more thoroughly. Indicate the significance level of the tests and whether the statistical results align with the expectations for a valid psychometric tool. Adding clarity on how these results support the psychometric validity of the scale would strengthen this section.
4. The conclusion in the abstract is appropriate, but it could be more concise by directly stating that the scale is not only psychometrically valid but also relevant for future interventions. It is important to emphasize how the study’s findings can lead to practical applications in HIV prevention.

**Introduction**

1. introduction presents various pieces of information (minority stress, HIV prevalence, etc.), but the connection between these elements and the study’s focus on HIV prevention behavior could be more explicitly stated. Ensure the flow logically progresses from the introduction of the problem (HIV among MSM) to the significance of the psychometric tool being studied.
2. The transition between the introduction of MSM as a vulnerable group and the prevalence of HIV among them could be more seamless. Focus on the significance of the HIV prevention behavior scale for MSM in Thailand specifically, and state why this research is necessary. The issue of outdated scales should be more directly tied to the need for an updated measure that aligns with current challenges and social contexts.
3. Concepts like the "minority stress model," "HIV prevention behavior," and other theories mentioned should be introduced with clearer definitions. Present these theories within the context of the study, making it clear why they are central to understanding the behavior of MSM in Thailand, specifically in relation to HIV prevention.
4. Although the introduction introduces relevant theories such as the Health Belief Model and Theory of Rational Action, it would benefit from a clearer integration of how these theories relate to the study's objectives. Expanding on the link between these theoretical frameworks and the HIV prevention behaviors in MSM would create a more robust narrative.
5. While the introduction mentions the absence of updated scales for HIV prevention behavior in Thailand, further emphasize the gap in the literature and how this study contributes to filling that gap. It would be helpful to discuss briefly why existing scales are inadequate in the context of Thai MSM and what this study hopes to improve or provide.

**Methods**

1. While the cross-sectional design is stated, more information is needed on why this design is suitable for the study’s objectives. For example, explain how cross-sectional data can be used to examine psychometric properties and why longitudinal data might not have been necessary for this particular research question.
2. The sampling method (snowball sampling) is described, but the rationale for using this method should be elaborated. Discuss the potential limitations of snowball sampling (e.g., selection bias, homogeneity of sample) and how these limitations are addressed. Moreover, clarify the justification for selecting individuals aged 25 and older, and explain if this age range reflects the target population for the HIV preventive behaviors scale.
3. The sample size calculation based on the Central Limit Theorem and the need for a minimum of 200 participants is mentioned but needs more explanation. Why specifically was this size chosen? It would be beneficial to present the power analysis or any other statistical justification for the sample size.
4. The section detailing the HIV Preventive Behavior Scale and other tools needs more specificity. For example, describe why the 9-item scale was developed, how the items were selected, and how they were related to the key HIV prevention behaviors. A more detailed explanation of the cognitive interview method would also be helpful, including the role of expert feedback and how this method enhanced the validity of the scale.
5. While the statistical methods (EFA, CFA, reliability analysis) are listed, more details should be provided about the specific steps and decisions made during these analyses. For instance, explain the criteria used for determining construct validity in the CFA, the thresholds for good model fit, and how these criteria relate to the goals of the study. Discuss how you handled potential issues like multicollinearity or cross-loadings in the factor analysis.
6. Provide more details about how participants’ consent was obtained, particularly in terms of the online survey. Discuss how anonymity was maintained, how the confidentiality of participants was ensured, and how the study complied with ethical guidelines for research with vulnerable populations.
7. How were participants recruited, and were there any specific challenges in obtaining a diverse sample? Also, describe how the data was stored and managed to ensure integrity.

**Results **

1. The categorization of the HIV-preventive behaviors into three broad groups (denial and avoidance, self-protective actions, and appropriate sexual response) should be more explicitly defined. The terms “denial,” “avoidance,” and “self-protective” need to be clearly operationalized, as their meaning could vary among different readers. For example, the term “self-protective” requires further elaboration to ensure that it is understood in a way that aligns with the research context. A more detailed explanation of how these categories were derived would improve clarity.
2. While the inclusion of participant quotes helps illustrate the themes, it would be beneficial to more systematically explain how these quotes were selected and their significance in relation to the overarching research questions. It is crucial to link the quotes directly to the themes identified in the categories (e.g., how does each participant’s quote specifically inform the "denial and avoidance" or "self-protective actions"?). Additionally, ensure that the formatting of these quotes is consistent, particularly when indicating direct statements.
3. The details of the factor analysis (e.g., PCA, EFA, CFA) should be more transparent. Specifically, while it is stated that "the first component was denial and avoidance of HIV risk," it is not clear how the decision was made to group certain items together in this category. More explanation should be given about the thresholds or criteria used for factor loadings and why certain items (like question 4) were moved between components. Additionally, clarify how you determined the adequacy of the model fit based on the indices presented (e.g., χ², GFI, RMSEA) and how these compare to established benchmarks in the literature.
4. The integration of demographic data with the psychometric analysis is lacking. To enhance the interpretation, demographic variables (such as age, sexual orientation, and frequency of sexual encounters) could be incorporated into the factor analyses or reliability tests to investigate whether certain variables influence the HIV preventive behavior scores.

**Discussion **

1.  The discussion should better articulate how the study's findings build upon or diverge from previous research. For instance, while references to studies by Pimthong and Bhanthumnavin are made, the specific ways in which this study either aligns or contrasts with their findings are not clearly outlined. You should highlight the significance of the two-factor model in this study and compare it in depth with similar models used in HIV prevention research, including contrasting findings from different cultural or regional contexts.
2. The two-component structure identified in the CFA (denial and avoidance, self-protective conduct) should be further examined in relation to the overall theory of HIV prevention behavior. How do these components relate to theoretical frameworks in sexual health and HIV prevention? Providing a more detailed theoretical underpinning for the categories would allow readers to better understand the broader implications of the findings. In particular, it would be valuable to explore whether these two components align with broader psychological theories of health behavior or HIV-related stigma.
3. The discussion should provide more practical recommendations for HIV prevention programs. While the findings are connected to previous studies, there is less focus on how the research results can inform interventions or policies targeting MSM. More concrete suggestions on how to integrate the two components of HIV prevention behavior into public health strategies would strengthen the relevance of the study.
4. The limitations section is briefly mentioned but should be expanded. It is essential to include a critical examination of the study’s limitations, such as the potential biases in the sampling process due to the use of online surveys, which could affect the generalizability of the findings. You should also discuss the limitations of relying solely on self-reported data, as this may introduce response biases, and the potential effects of social desirability or recall bias on the results. Additionally, the issue of sample homogeneity in terms of age or sexual orientation should be discussed, as it might limit the applicability of the findings to broader populations.
5. While the limitations section touches on some areas for improvement, the discussion should provide a more thorough outline of potential future research. Consider recommending further studies that examine the cultural nuances in HIV preventive behaviors among MSM in other regions or countries. Also, explore how different prevention interventions might be tailored to the specific behaviors identified in this study, such as targeting denial and avoidance or enhancing self-protective behaviors.
6. In line with the limitations, it would be beneficial to suggest methodological refinements for future studies, such as the inclusion of qualitative interviews to gain deeper insights into the underlying psychological mechanisms behind the two components identified. This would complement the quantitative data and provide a more comprehensive understanding of MSM’s HIV prevention behaviors.
7. The discussion would benefit from a more in-depth exploration of the cultural context within Thailand, and how it might influence the reported HIV preventive behaviors. For instance, consider discussing how societal views on sexuality, masculinity, and HIV risk might shape the denial/avoidance and self-protective behavior categories identified in this study. This would provide a more nuanced understanding of the findings and their implications for HIV prevention within this specific cultural setting.

Is the work clearly and accurately presented and does it cite the current literature?

No

If applicable, is the statistical analysis and its interpretation appropriate?

No

Are all the source data underlying the results available to ensure full reproducibility?

No

Is the study design appropriate and is the work technically sound?

Partly

Are the conclusions drawn adequately supported by the results?

No

Are sufficient details of methods and analysis provided to allow replication by others?

No

Reviewer Expertise:

MindfulnessAdolescent Mental HealthStressEmotionCyberpsychology

I confirm that I have read this submission and believe that I have an appropriate level of expertise to state that I do not consider it to be of an acceptable scientific standard, for reasons outlined above.
